# Membrane vesicles from oral pathobiont *Porphyromonas gingivalis* and oral commensal *Neisseria oralis* both induce Tau phosphorylation but different murine brain responses

**DOI:** 10.1002/alz70855_104710

**Published:** 2025-12-24

**Authors:** Giuseppe D Ciccotosto, Elly Bijlsma, Stuart G Dashper, Catherine A Butler

**Affiliations:** ^1^ The University of Melbourne, Parkville, VIC, Australia

## Abstract

**Background:**

We have previously shown that repeated oral inoculation of mice with bacteria that cause the chronic oral disease periodontitis resulted in brain infiltration and induction of Alzheimer's disease (AD)‐like pathology. Here, we examined whether purified bacterial membrane vesicles (BMVs) from the oral pathobiont *Porphyromonas gingivalis* or oral commensal *Neisseria oralis* could enter the brain and cause pathology, without the need for an infection of the brain.

**Methods:**

Three experimental groups of 36 female C57BL/6 mice, were injected intravenously with 10 µg of purified BMVs from *P. gingivalis* or *N. oralis*, or an equal volume of vehicle alone (Control), through the tail vein on Days 1, 5, 8 and 11. Six mice from each group were killed on Days 2, 6, 12, 26, 40 and 55, then PBS perfused before their brains dissected. Immunohistochemistry analyses of 3 hemibrains per group, per timepoint, were probed for the presence of the BMVs, neuronal damage, neuroinflammation, AD‐like pathology and immune system activation.

**Result:**

BMVs from both *P. gingivalis* and *N. oralis* were found within the brains after one injection. Detection of *P. gingivalis* MV positive cells peaked at two timepoints, at Days 6 and 26, whereas detection of *N. oralis* MV positive cells peaked at Day 26, 2 weeks after the final intravenous injection. By Day 55, the detection of cells positive for both BMVs had decreased to levels consistent with the BMVs being removed from the brains. There were similar levels of neuronal degeneration, Il‐6 and IL‐1β in the hippocampus between the three groups of mice over time, but only *P. gingivalis* MVs were able to induce microgliosis and astrogliosis in the hippocampus. There were no amyloid plaques detected in the brains, but phosphorylated Tau peaked significantly at Day 26 in the mice that received BMVs.

**Conclusion:**

BMVs from both *N. oralis* and *P. gingivalis* entered the brain from the bloodstream but caused different host responses. Upon cessation of BMV injection, the BMVs were naturally removed from the brain over time, suggesting that treatment of the source of the BMVs, namely periodontitis, would reduce exposure to brain pathology causing agents.

